# Treatment of Flue Gas Desulfurization Wastewater by an Integrated Membrane-Based Process for Approaching Zero Liquid Discharge

**DOI:** 10.3390/membranes8040117

**Published:** 2018-11-26

**Authors:** Carmela Conidi, Francesca Macedonio, Aamer Ali, Alfredo Cassano, Alessandra Criscuoli, Pietro Argurio, Enrico Drioli

**Affiliations:** 1 Institute on Membrane Technology, ITM-CNR, c/o University of Calabria, via P. Bucci, 17/C, I-87036 Rende (CS), Italy; c.conidi@itm.cnr.it (C.C.); amir_hmmad@hotmail.com (A.A.); a.criscuoli@itm.cnr.it (A.C.); e.drioli@itm.cnr.it (E.D.); 2 Department of Environmental and Chemical Engineering, University of Calabria, Via P. Bucci, 44/A, I-87036 Rende (CS), Italy; pietro.argurio@unical.it

**Keywords:** FGD wastewater, integrated membrane-based process, zero liquid-discharge, sustainability

## Abstract

An integrated membrane process for the treatment of wastewaters from a flue gas desulfurization (FGD) plant was implemented on a laboratory scale to reduce their salt content and to produce a water stream to be recycled in the power industry. The process is based on a preliminary pretreatment of FGD wastewaters, which includes chemical softening and ultrafiltration (UF) to remove Ca^2+^ and Mg^2+^ ions as well as organic compounds. The pretreated wastewaters were submitted to a reverse osmosis (RO) step to separate salts from water. The RO retentate was finally submitted to a membrane distillation (MD) step to extract more water, thus increasing the total water recovery factor while producing a high-purity permeate stream. The performance of RO and MD membranes was evaluated by calculating salts rejection, permeate flux, fouling index, and water recovery. The investigated integrated system allowed a total recovery factor of about 94% to be reached, with a consequent reduction of the volume of FGD wastewater to be disposed, and an MD permeate stream with an electrical conductivity of 80 μS/cm, able to be reused in the power plant, with a saving in fresh water demand.

## 1. Introduction

Much research is today focusing on minimizing the scarcity of potable water and the impact of air, water, and solid waste pollutants. Among them, to improve the control of SO_2_ emissions, various flue gas desulfurization (FGD) technologies have been developed in the last decades.

Flue gas desulfurization processes are primarily used to remove SO_2_ from exhaust flue gases of fossil fuel thermoelectric power plants. In these plants, SO_2_ is produced during the combustion of coal and oil, and can be further converted (about 1%) into sulfur trioxide (SO_3_) if high contents of oxygen are present [1]. The commonly used FGD technologies are: Wet and spray-dry scrubbing (using a slurry of alkaline sorbents, like limestone or lime, or seawater); the wet sulfuric acid process (recovering sulfur as sulfuric acid); and dry sorbent injection systems.

Depending on the coal source, used technology, and operating conditions, FGD processes can give origin to various streams, with a different and complex composition. Chloride, sulfate, nitrate, calcium, magnesium as well as various heavy metals and dissolved silica and borate are often present. Moreover, FGD wastewater may have a content of total dissolved solids (TDS) as high as 50,000 mg/L [2].

The treatment of FGD wastewater represents one big issue in the water industry [2,3]. One possibility is to use biotechnological treatments, which, however, lead to the production of H_2_S [4].

Another option consists in applying chemical precipitation-based strategies for heavy metal removal, which use alkaline compounds, such as Ca(OH)_2_ (hydrated lime), NaOH (caustic soda), or Na_2_CO_3_ (soda ash), leading to the production of insoluble hydroxides. This approach is limited by the production of huge quantities of heavy metal hydroxide and calcium sulfate sludge that have to be disposed in a regulated hazardous waste facility.

The use of zero-valent iron (ZVI or Fe0) as reactive media for treating heavy metal contaminated groundwater has also been investigated in the last years [5,6,7,8]. The addition of iron promotes the removal of dissolved heavy metals by several mechanisms, including cementation, precipitation of metal hydroxides, and adsorption [9]. This approach has also been evaluated for the removal of selenium [10,11], mercury, and other heavy metals [12] from FGD wastewaters. In particular, continuous-flow field tests conducted on a fluidized bed system consistently reduced Hg from ca. 50 to <0.005 μg/L and Se (mostly as selenate) from ca. 3000 to <7 μg/L. Most of the heavy metals were all reduced to near- or sub-ppb levels [12]. However, the potential of ZVI as a reagent for remediating contaminated FGD wastewaters is limited by the rapid loss of ZVI reactivity upon the formation of iron corrosion products as a passive coating on the ZVI grains [13].

In recent years, the potential of membrane operations, like microfiltration (MF), ultrafiltration (UF), nanofiltration (NF), and reverse osmosis (RO), for treatment of different wastewaters has been investigated. The attraction of membrane operation was, among others, due to their capability of removing almost all pollutants with a reduced addition of chemicals (only the amount necessary for membrane pre-treatment and cleaning).

Apart from chemical and biotechnological processes, membrane operations can be a viable approach for the remediation of FGD wastewaters, although few studies have been reported until now on this subject. The performance of a coprecipitation method of heavy metal hydroxides and sulphides followed by crossflow microfiltration (CMF) in the treatment of wastewater from a FGD plant was analyzed by Enoch et al. [14]. The removal efficiency of both hydrophilic and hydrophobic membranes was satisfactory, except for Cd removal.

Liu et al. [15] also used MF membranes, with and without an initial chemical precipitation, for the removal of Hg^2+^ from FGD wastewater, demonstrating the feasibility of the process.

An integrated membrane system, constituted of a sedimentation tank for particles sedimentation, UF, NF, and RO, was utilized by Yin et al. [16] for the treatment of the desulfurization wastewater. In particular, the sedimentation step revealed to be fundamental to improve UF flux and permeate quality. As a matter of fact, without pre-sedimentation, a lower steady flux of 200 L/m^2^h was reached, compared to about 500 L/m^2^h achieved in the UF process with pre-sedimentation, at a transmembrane pressure (TMP) of 0.15 MPa, temperature of 40 °C, and cross-flow velocity (CFV) of 4 m/s. UF retained 99.9% of the initial suspended sulfur (SS) while salt compounds passed through the UF membrane and were recovered in the permeate. Therefore, UF ceramic membranes were considered for removing SS, generating a permeate stream able to be treated by the NF unit. The NF unit separated bivalent ammonium salts from monovalent ammonium salts and the obtained NF permeate was sent to RO to separate monovalent salts from water, and to recover ammonium thiocyanate. The analyzed integrated membrane process (UF/NF/RO) was able to reduce environmental loads and to make possible the recovery of various valuable substances (such as, sulfur and NH_4_SCN) and the water reuse.

Being a pressure driven membrane operation that is well consolidated also at the industrial level, RO has the great advantage of producing high quality water (rejection factor higher than 99% for monovalent and bivalent ions in the feed) at a relatively low energy consumption (3 and 4 kWh/m^3^ are typical values at around a 50% recovery factor). The main RO limitation is the high feed pressure required for overcoming the feed side osmotic pressure, which usually ranges from 55 to 68 bar for a salinity of about 35g/L, and that is around 15 bar for salinity between 0.5 and 30 g/L [17]. Being a thermally driven membrane separation technology, in membrane distillation (MD), operating pressures are generally on the order of a few hundred kPa. Moreover, its performance is less affected by concentration polarization than pressure driven membrane operations, allowing the treatment of higher salinity water and leading to a higher recovery factor, then reducing the environmental impact of the produced brine and with the potential to achieve near zero discharge. In fact, various studies proved that MD could accomplish nearly complete salt rejection and high water reclamation in the treatment of high-salinity wastewater [18,19,20,21], as is the case of FGD wastewater produced by seawater scrubbing. Moreover, MD has the additional advantage of a relatively low operating temperature, with the possibility to utilize either waste heat or renewable energy resources (such as geothermal or solar energy) or the low-grade heat available in power plants [22,23,24].

Jia and Wang [25] recently investigated an integrated process for the treatment of flue gas desulfurization wastewater based on chemical softening followed by NF and MD working on the NF permeate. They found that the chemical softening and the NF pretreatment could significantly decrease membrane scaling in MD. Moreover, over 99.99% salt rejection and over 92% of water reclamation were achieved. The MD configuration used by authors was the vacuum membrane distillation (VMD) because of its high flux. Nevertheless, this configuration needs the use of a vacuum pump and of an external condenser for the permeate recovery.

**Figure 1:**
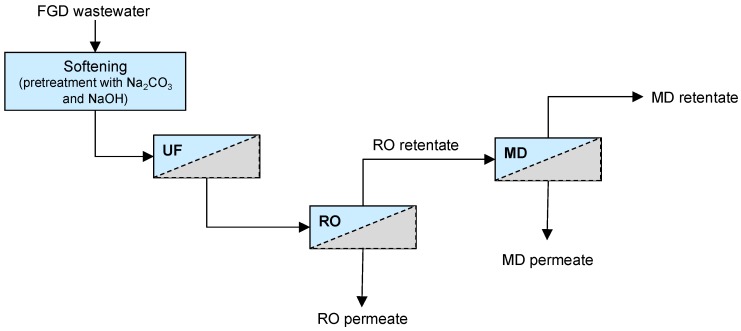
Scheme of the integrated membrane system investigated.

In a previous work, we evaluated the performance of two commercial RO spiral-wound membranes (SWC-2540 and ESPA-2540 both from Hydranautics) for the removal of salt compounds from softened and ultrafiltered FGD wastewaters [26]. The SWC membrane was more effective than the ESPA membrane in terms of ions rejection, fouling index, and cleaning efficiency, and a permeate conductivity lower than 2 mS/cm was obtained. Based on the positive results achieved, in the current study, we investigated the possibility to treat the RO brine produced by a SWC-2540 RO membrane in an MD unit, with the aim of producing higher purity water as MD permeate, while reducing the brine volume to be disposed. As in the previous study [26], FGD wastewaters were submitted to a pre-treatment step (softening and UF) aimed at reducing Ca^2+^ and Mg^2+^ content, as well as the organic content, to minimize scaling. The UF permeate was then processed by the SWC-2540 RO membrane and its brine was finally submitted to an MD step (Figure 1). Direct Contact Membrane Distillation (DCMD) was chosen because, among the MD configurations, it is the simplest to operate, requiring the least equipment.

## 2. Materials and Methods

### 2.1. Pretreatment of FGD Wastewaters

FGD wastewaters were collected from the thermal power plant of Industrial Enel Research (Brindisi, Italy). The whole pretreatment process consisted of softening agents’ addition followed by UF.

FGD wastewaters were softened with Na_2_CO_3_·H_2_O (Na_2_CO_3_·H_2_O/Ca molar ratio 2.6/1) and NaOH (NaOH/Mg molar ratio 1.5/1); the solutions were incubated for 1h at room temperature and then pre-filtered with filter paper (pore size of about 10–20 μm) before acidification with H_2_SO_4_ up to pH 6.5. Afterwards, the FGD wastewaters were treated with an antiscalant (Carboxyline CM supplied by Aquastill B.V., Sittard, The Netherlands) at the recommended concentration (8 mg/L) in order to prevent the precipitation of low soluble salts.

Cross-flow UF was performed by using a hollow fiber polyethersulphone (PES) membrane module (FUS 5082, Microdyn Nadir, Wiesbaden, Germany), having a nominal molecular weight cut-off (MWCO) of 500 kDa and an effective membrane area of 0.25 m^2^. A feed-and-bleed configuration (in order to work at constant feed volume, during the experiments feed was added in the feed tank in the same amount of the produced permeate) was operated until a recovery factor (RF) of 98% was obtained at the following experimental conditions: TMP of 0.45 bar, axial feed flowrate (Q_f_) of 560 L/h, and temperature (T) of 25 ± 1 °C. In the selected operating conditions, average permeate fluxes in the range of 460–500 kg/m^2^h were registered.

After the treatment with FGD wastewater, the UF membrane module was rinsed with distilled water for 30 min and then cleaned by recycling an acid detergent (Ultraclean WO, Henkel Chemicals Ltd., Dusseldorf) at a concentration of 0.1% (w/w) (pH 4) for 60 min at 40 °C. After, the membrane module was rinsed with distilled water for 20 min. The cleaning-in-place procedure allowed more than 98% of the initial permeability of the UF membrane to be recovered.

### 2.2. RO and MD: Experimental Set-Up and Membranes

RO experiments were carried out using the laboratory set-up of Matrix Desalination Inc. (Florida, USA), which incorporated a stainless steel housing able to accommodate a 2.4 × 21 in. spiral-wound membrane module with an effective membrane surface area of 2.34 m^2^ (main properties are listed in Table 1). The equipment consists of a feed tank with a capacity of 20 L, a high-pressure pump, a back-pressure valve, two pressure gauges, a permeate control valve, and a coiling cool fed with tap water used to maintain the control the feed temperature.

The pretreated FGD wastewaters were treated in a spiral-wound RO membrane module (SWC-2540) supplied by Hydranautics Corporation (Oceanside, CA, USA), whose properties are summarized in Table 1. Experiments were performed according to a feed-and-bleed configuration in selected operating conditions (TMP, 36 bar; Q_f_, 204 L/h; T, 26.5 °C) up to an RF of 60%.

The permeate flux was determined by weighing the amount of permeate collected vs. time and using the following equation:

(1) $$\;J_{p}=\frac{m_{p}}{A\cdot t}\;$$

where *J_p_* is the permeate flux (kg/m^2^h), *m_p_* the permeate weight (kg) at time, *t* (h), and *A* is the membrane surface area (m^2^).

The water permeability (*W_p_*) of the membrane was obtained as the slope of the straight line resulting from plotting the water flux at 25 °C versus the applied TMP.

After each experiment, the RO membrane was rinsed with water at 30 °C and then cleaned with an acid solution (Ultraclean WO 0.05%, pH 4) at 40 °C, for 60 min. Then, the membrane was rinsed with distilled water for 20 min and the water permeability was remeasured. 

The fouling index of the RO membrane (*FI_RO_*) was calculated by the following equation:

(2) $$\;FI_{RO}=\left(1-\frac{W_{p1}}{W_{p0}}\right)\cdot 100\;$$

where *W_p0_* and *W_p1_* are the water permeability measured before and after the FGD wastewater treatment. 

The cleaning efficiency was evaluated by using the water flux recovery method, according to the following equation:

(3) $$\;CE=\left(\frac{W_{p3}}{W_{p0}}\right)\cdot 100\;$$

where *W_p3_* is the water permeability measured after the chemical cleaning.

Membrane distillation was performed in a direct contact configuration (DCMD). In the lab plant, the MD feed (i.e., RO retentate) and the MD permeate streams (i.e., demineralized water) converge in the counter-current mode towards the membrane module containing the flat oleophobic membrane supplied by Aquastill. The main properties of the used membrane are summarized in Table 2.

The driving force in DCMD is a vapour pressure difference across the membrane, which is imposed by a temperature difference across the membrane. Therefore, the retentate line was heated by an ISCO GTR 2000 heater (Isco srl, Fizzonasco di Pieve Emanuele (MI), Italy) whilst the permeate line was cooled by a RTE 17 NESLAB refrigerated bath chiller circulator (Thermo Electron Corporation, Newington, CT, USA). The retentate solution and distillate coming out from the module were returned back to the feed tank and permeate tank, respectively, both working at atmospheric pressure. Magnetic drive gear pumps (Iwaki Co., Ltd., Tokyo, Japan) were used to recirculate the streams. The plant was also equipped with flow meters, thermocouples, manometers, and a conductivity meter.

The trans-membrane flux was calculated by evaluating weight variations in the distillate tank by a Gibertini EU-C LCD balance (Gibertini Elettronica, Novate Milanese (MI), Italy). After each experiment, the MD membrane was rinsed with water. The fouling index of the MD membrane (*FI_MD_*) was calculated by:

(4) $$\;FI_{MD}=\left(1-\frac{J_{w1}}{J_{w0}}\right)\cdot 100\;$$

where *J_w0_* and *J_w1_* are the MD trans-membrane flux before and after RO retentate treatment, respectively, when distilled water is used as the MD feed and the membrane process is carried out at 48 °C.

On the basis of previous MD experiments directly performed on FGD wastewaters [29], experimental runs were carried out at the operative conditions reported in Table 3.

### 2.3. Analytical Measurements

The different samples collected from the investigated processes were analyzed for electrical conductivity (EC), total dissolved solids (TDS), Ca^2+^, Mg^2+^, Na^+^, TOC, and pH.

The removal efficiency for each component was expressed in terms of rejection (R) according to the following equation:

(5) $$\;R=1-\left(\frac{C_{p}}{C_{f}}\right)\cdot 100\;$$

where *C_f_* and *C_p_* are the concentrations of a specific component in the feed and permeate, respectively.

EC and TDS were measured using a digital conductivity meter (HI 2300 Microprocessor Conductivity, Hanna Instruments, Woonsocket, RI, USA).

Ca^2+^, Mg^2+^, and Na^+^ concentrations were determined by using a high-resolution continuum source atomic absorption spectrometer (HR-CSAAS, ContrAA700, Analytik Jena AG, Jena, Germany), with a high intensity Xe short arc lamp as a continuum source. Samples and standards were appropriately diluted (300 times for Mg and Ca, 3000 times for Na). Subsequently, they were acidified with 1% HCl and the absorbance measurements were performed using the spectral lines at 422.67 nm, 588.99 nm, and 285.21 nm for Ca^2+^, Na^+^, and Mg^2+^, respectively.

pH was measured by an Orion Expandable ion analyzer EA 920 pH meter (Allometrics, Inc., Baton Rouge, LA, USA) with automatic temperature compensation.

Total organic carbon (TOC) was analyzed by a TOC analyzer (TOC-V CSN, Shimadzu, Kyoto, Japan).

## 3. Results and Discussion

### 3.1. Pretreatment of FGD Wastewaters

The chemical composition of FGD wastewater before and after the pre-treatment step is reported in Table 4. Raw waters were characterized by a lower content of Ca^2+^ and Mg^2+^ when compared to typical FGD wastewaters sampled by United States Environmental Protection Agency (USEPA) in several US thermal plants (Ca^2+^ in the range of 2000–5400 ppm and Mg^2+^ in the range of 1000–4200 ppm, respectively) [3]. Indeed, the concentration of Ca^2+^ and Mg^2+^ before the pre-treatment was approximately 384.4 ± 4.8 ppm and 289.9 ± 2.6 ppm, respectively. On the other hand, the average content of Na^+^ in the raw water was significantly higher (7.28 ± 0.6 g/L) than that found in typical wastewaters sampled by USEPA (50–2000 mg/L) [3]. These differences can be attributed to several aspects, which contribute to the pollutant concentrations of FGD wastewaters, including the coal type, the sorbent used, the materials of construction in the FGD system, and the FGD system operation. The addition of sodium carbonate and sodium hydroxide in the conditions previously optimized [26] permitted the content of Ca^2+^ and Mg^2+^ of raw wastewaters to be reduced, with a removal efficiency of 90.0% and 78.8%, respectively. A lower removal efficiency was measured for Na+ (3.84%). The chemical pre-treatment allowed the achievement of a 7.4% removal of TDS (from 16.9 ± 0.6 g/L to 15.7 ± 0.8 g/L); a further removal was reached after the UF treatment, with a final overall value of 13.49%. The UF process slightly changed the concentrations of the analysed compounds if compared with the chemical pre-treated FGD wastewater, as it could be expected from the MWCO of the used UF membrane. Nevertheless, the UF step removed more than 60% of TOC. This rejection value can be explained assuming the presence of not totally dissolved organic solids forming micro-droplets with a size in the range of the MWCO of the UF membrane.

### 3.2. Reverse Osmosis and Membrane Distillation Performance

**Figure 2:**
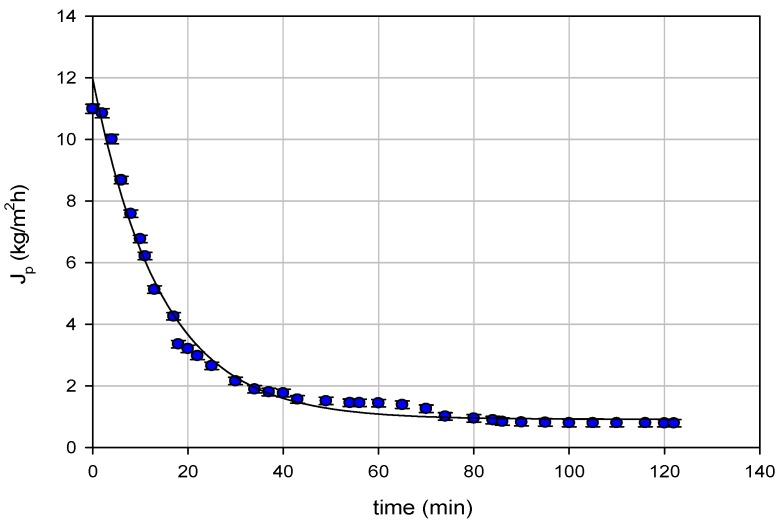
Reverse osmosis (RO) of pre-treated FGD wastewaters. Time course of permeate flux. (TMP = 36 bar; T = 26.5 °C; Q_f_ = 204 L/h).

The RO membrane performance was assessed in terms of the permeate flux (*J_p_*), membrane cleaning efficiency, and salt removal efficiencies. The time evolution of the permeate flux of pre-treated wastewaters in selected operating conditions is illustrated in Figure 2. Experimental data are referred to the processing of 16.1 kg of pre-treated FGD wastewaters with a production of 10.1 kg of permeate (recovery of about 63%). The initial permeate flux of 11 kg/m^2^h sharply decreased during the first 20 min, then continued to reduce in time, reaching a pseudo-steady state value higher than 1 kg/m^2^h after 120 min. The permeate flux decline can be due to different factors, like the increase of the osmotic pressure during continuous concentration of the feed solution, as well as to membrane fouling and concentration polarization phenomena [30]. In particular, concentration polarization leads to a higher solute concentration at the membrane surface, which may cause salts deposition and also high osmotic pressure at the membrane surface, which means a flux decline when RO is performed at constant pressure [31].

**Figure 3:**
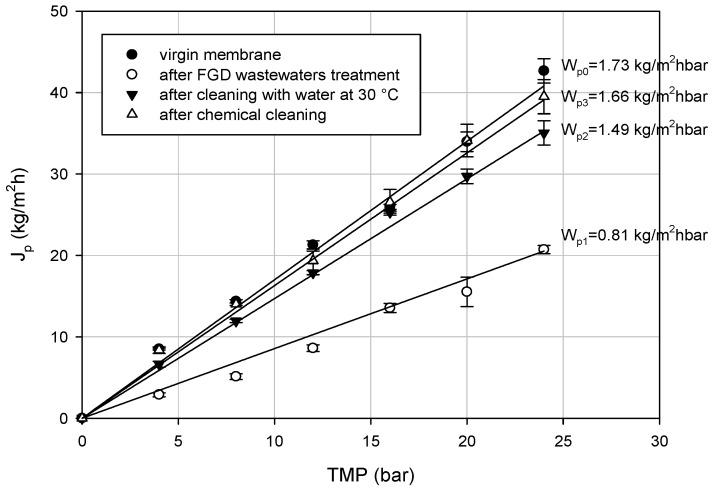
Permeate flux variation with TMP for the RO membrane before and after cleaning procedures (*W_p_*_0_ initial hydraulic permeability; *W_p_*_1_ hydraulic permeability after RO treatment; *W_p_*_2_ hydraulic permeability after cleaning with water; *W_p_*_3_ hydraulic permeability after cleaning with acid detergent).

Figure 3 shows the hydraulic permeability of the RO membrane before and after the treatment of the UF permeate and after cleaning procedures: The initial hydraulic permeability (*W_p_*_0_) of about 1.73 kg/m^2^hbar was reduced to 0.81 kg/m^2^hbar after the treatment of the UF permeate. According to these data, the fouling index was about 53.1%. A first cleaning with distilled water at 30 °C recovered about 86% of the initial permeability (*W_p_*_2_ =1.49 kg/m^2^hbar); a higher water flux recovery (of about 96%) was reached through the chemical cleaning with acid detergent (*W_p_*_3_ = 1.49 kg/m^2^hbar). Therefore, experimental data confirmed that acid solutions are effective in removing membrane scaling of RO membranes [32].

**Figure 4:**
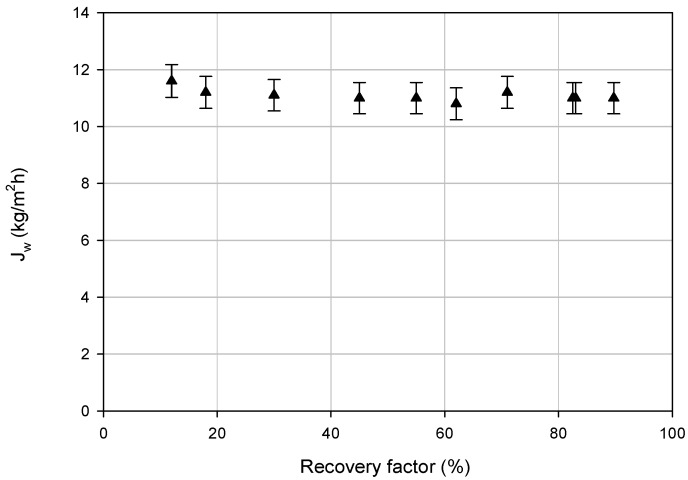
MD trans-membrane flux vs recovery factor.

The RO retentate was further concentrated by DCMD. The trend of the transmembrane flux (*J_w_*) as a function of the MD recovery factor is reported in Figure 4. Experimental results indicated that the MD flux was not significantly affected by the recovery factor despite the growing feed concentration. The measured average flux was about 11 kg/m^2^h. Additionally, the MD water recovery factor was also excellent and equal to 89.7%. Moreover, the quite constant trend of the trans-membrane flux indicated that no significant fouling occurred in the MD test. This was confirmed by the value of FI_MD_ (1.76%) determined via Equation (3).

These results were in agreement with those reported by Jia and Wang [25] in the treatment of FGD wastewaters by NF and MD. In their approach, the authors employed chemical softening and NF as pretreatment followed by VMD. The membrane flux of the VMD process remained stable during the whole continuous concentration and was of the order of 8.5 L/m^2^h. On the other hand, the flux of a direct VMD process, which employed only MF as pretreatment, decreased sharply in the first 6h due to the formation of CaSO_4_.

The chemical composition of the different samples coming from the RO/MD integrated process is summarized in Table 5. The EC of FGD wastewaters in the RO permeate was lowered down to 5.08 ± 0.10 mS/cm, with a removal efficiency of 85.4%.

**Figure 5:**
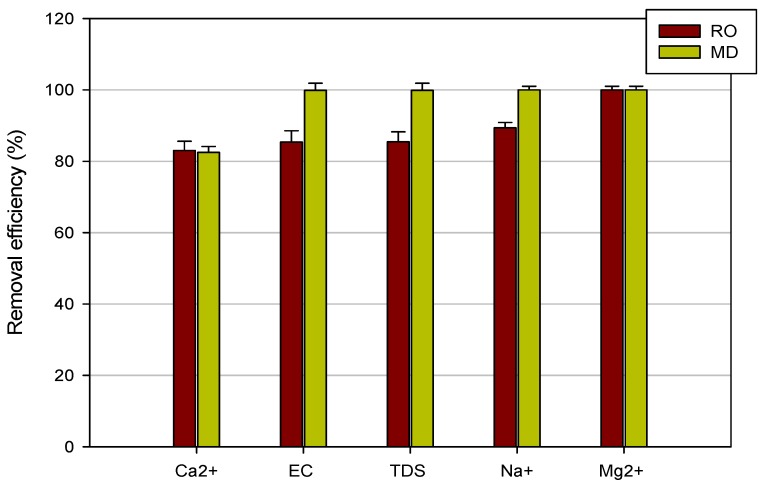
Removal efficiency of RO and MD membranes towards analyzed compounds.

TDS, Ca^2+^, and Na^+^ ion concentrations in the RO permeate strongly reduced when compared with the feed solution. The observed rejection of the RO membrane towards Na^+^ was of about 90%, whereas, for Ca^2+^, TDS, and EC, rejections were higher than 83%. Mg^2+^ ions were completely removed by the RO membrane, with a removal efficiency of 100% (Figure 5).

The MD step removed both Mg^2+^ and Na^+^ ions from the RO retentate (Figure 5). TDS and EC rejections were also very high and equal to 99.9%. A lower retention value was measured for Ca^2+^ ions (about 82.5%). This lower rejection could be attributed to calcium scaling on the membrane, which, however, did not affect the transmembrane flux (see Figure 4).

According to the experimental results, the combination of RO and MD processes reached a total recovery factor of about 94% and an MD permeate with a quality standard suitable to be reused in the power plant (needed purity: EC < 800 μS/cm), with a consequent saving in fresh water consumption and reduction of the volume of FGD wastewater to be disposed.

## 4. Conclusions

In the last decade, various flue gas desulfurization (FGD) technologies have been developed for removing sulfur dioxide from flue gases coming from fossil fuel thermoelectric power plants. FGD processes give origin to high salinity streams with a complex composition and the efficient treatment of FGD wastewater is one of the biggest challenges today.

In the present work, the problems of reducing water demand in power plants and of minimizing FGD wastewater discharge were dealt with. For this purpose, the potential of an integrated membrane-based process for FGD wastewater treatment and reuse was evaluated. In particular, the lab scale plant included: (1) A pre-treatment (chemical softening and UF) for reducing Ca^+2^, Mg^2+^, and TOC concentration in the raw wastewater, (2) an RO unit for the separation of salts from water, and (3) a MD unit for the treatment of RO retentate to extract more water, thus increasing the total water recovery factor of the process.

The experimental results indicated that high quality RO and MD permeate streams were obtained. In particular, the integrated RO/MD process achieved a total recovery factor of about 94% and an MD permeate stream with an electrical conductivity of 80 μS/cm that makes it suitable to be reused in the power plant. This will imply significant benefits in terms of a reduction of water demand in the plant, minimization of wastewater to be discharged in the environment, and overall improvement in the sustainability of the process.

## Figures and Tables

**Table 1:** Characteristics of the reverse osmosis (RO) membrane module.

**Membrane Type**	SWC-2540
**Membrane Material**	Composite polyamide
**Configuration**	Spiral-wound
**Salt Rejection (%)**	99.4 (minimum 99.0)
**pH Operating Range**	2–11
**Max. Operating Temperature (°C)**	45
**Max. Operating Pressure (bar)**	69
**Membrane Surface Area (m^2^)**	2.34
**Water Permeability (kg/m^2^hbar)**	1.77 ^a^
**Zeta Potential (mV)**	−21.2 at pH 7 ^b^
**Contact Angle**	96.05 ± 4.35 ^c^

^a^ our data; ^b^ data from Li et al. [27]; ^c^ data from Lee et al. [28].

**Table 2:** Characteristics of the oleophobic membrane used in membrane distillation (MD).

**Membrane Material**	Polyethyelene-oleophobic (PE-O)
**Configuration**	Flat sheet
**Active Module Length**	50 cm
**Membrane Area**	0.05 m^2^
**Mean Pore Size**	0.3 μm
**Porosity**	80%
**Membrane Thickness**	76 μm
**Liquid Entry Pressure (LEP)**	>4 bar
**Contact Angle**	>118°

**Table 3:** MD operative conditions.

**MD Feed Solution**	RO brine
**T_Feed, in_, °C**	69 ± 0.1
**T_Permeate, in_, °C**	28 ± 0.3
**Feed Flow Rate, l/min**	0.5
**Permeate Flow Rate, l/min**	0.4

**Table 4:** Chemical composition of flue gas desulfurization (FGD) wastewater before and after pre-treatment.

Parameter	Sample			Overall Removal (%)
	Raw Water	After Softening	After UF	
Ca^2+^ (ppm)	384.4 ± 4.8	39.16 ± 2.1	38.13 ± 2.1	90.00
Mg^2+^ (ppm)	289.9 ± 2.6	62.5 ± 0.5	62.4 ± 0.1	78.84
Na^+^ (g/L)	7.28 ± 0.6	7.0 ± 0.14	7.0 ± 0.3	3.84
EC (mS/cm)	33.6 ± 2.1	32.5 ± 1.2	31.1 ± 1.7	7.44
TDS (g/L)	16.9 ± 0.6	15.7 ± 0.8	14.62 ± 1.2	13.49
TOC (mg/L)	-	90.12 ± 0.90	33.80 ± 0.34	62.50
pH	6.7 ± 0.1	6.55 ± 0.2	6.8 ± 0.1	-

**Table 5:** Chemical composition of FGD wastewaters coming from RO/MD treatments.

Sample	Ca^2+^ (ppm)	Mg^2+^ (ppm)	Na^+^ (g/L)	EC (mS/cm)	TDS (g/L)	pH
Feed RO	40.1 ± 0.8	67.4 ± 1.3	6.9 ± 0.1	34.7 ± 0.7	17.4 ± 0.9	7.53 ± 0.4
Permeate RO	6.9 ± 0.4	n.d.	0.70 ± 0.01	5.08 ± 0.10	2.53 ± 0.10	7.12 ± 0.14
Retentate RO	92.7 ± 1.8	175.1 ± 3.5	15.9 ± 0.3	69.4 ± 1.4	35.8 ± 0.7	7.78 ± 0.15
Permeate MD	16.22 ± 0.32	n.d.	n.d.	0.080 ± 0.001	0.040 ± 0.001	6.37 ± 0.13
Retentate MD	286.64 ± 5.37	539.8 ± 10.8	4.9 ± 0.1	158.3 ± 3.1	78.8 ± 1.6	8.15 ± 0.16

n.d.: not detectable.
